# Knowledge, attitudes, and practices towards prevention and control of neurocysticercosis-related epilepsy in Northern Tanzania: A cross-sectional healthcare professional-based study

**DOI:** 10.1371/journal.pntd.0013275

**Published:** 2025-08-01

**Authors:** Masanja Laurent Lugelongi, Andrew Martin Kilale, Kabemba Evans Mwape

**Affiliations:** 1 Department of Clinical Studies, School of Veterinary Medicine, The University of Zambia, Lusaka, Zambia; 2 Livestock Training Agency (LITA), Tengeru Campus, Ministry of Livestock and Fisheries, Arusha, Tanzania; 3 National Institute for Medical Research, Muhimbili Research Centre, Dar es Salaam, Tanzania; Seoul National University College of Medicine, KOREA, REPUBLIC OF

## Abstract

**Background:**

Infection of the brain with the larval stage of *Taenia solium* leads to neurocysticercosis (NCC), a leading cause of epilepsy with a considerable global burden. Neurocysticercosis-induced epilepsy is among the poverty-related diseases and is prevalent, particularly in regions where *T. solium* is endemic. In Tanzania, NCC-associated epilepsy is endemic, it causes substantial health burdens and economic losses. Studies on knowledge, attitudes, and practices (KAP) regarding NCC in the country are limited.

**Methodology:**

A cross-sectional healthcare professional-based study was conducted in northern Tanzania between May and July 2023. A structured questionnaire was administered to healthcare workers (HCWs) using a random stratified sampling to assess their knowledge, attitudes, and practices toward prevention and control of NCC-induced epilepsy. One-way ANOVA was used to compare mean scores of KAP, Chi-square test to assess the association between independent factors and KAP scores, and logistic regression model to determine the predictors of KAP scores.

**Results:**

A total of 250 HCWs were interviewed. The study revealed that HCWs had inadequate overall knowledge (scores = 55.5%), negative attitudes (score = 54.0%) and poor practices (scores = 49.3%). Only 22.4% had adequate knowledge, 10.8% had positive attitudes, and 12.8% had good practices towards control of NCC-induced epilepsy. Educational levels of respondents (OR: 3.361, 95% CI: 1.147–9.848, p < 0.01) were significant predictors of positive attitudes. Laboratory technologists were five (OR:5.821, 95% CI: 2.033-16.664, p < 0.01) times more likely to be knowledgeable, followed by assistant physicians (OR:3.141, 95% CI:1.484-6.650, p < 0.01) compared to other health professionals interviewed. Knowledgeable respondents were more likely to have positive attitudes (OR: 6.087, 95% CI:2.534-14.622, p < 0.01), and better practices (OR: 4.750, 95% CI: 2.099-10.748, p < 0.01) towards control of NCC-induced epilepsy.

**Conclusion:**

This study found that HCWs had limited knowledge, poor practices, and negative attitudes toward the control of NCC-induced epilepsy. There is an urgent need to promote better knowledge of acquired epilepsy among HCWs, including re-training using the One Health approach in *T. solium* endemic areas.

## Author summary

*Taenia solium* neurocysticercosis (NCC) is the main cause of preventable acquired epilepsy particularly in endemic settings Tanzania included. The study aimed to assess the knowledge, attitudes and practices (KAP) of healthcare workers (HCWs) in the primary health facilities, towards control and prevention of NCC-induced epilepsy in northern Tanzania. Early detection of tapeworms’ carries and treatment using effective anthelmintics (albendazole, praziquantel) are ideal for interrupting the lifecycle of the *T. solium,* and eventually control and prevent NCC-induced epilepsy. Our study established knowledge gaps, negative attitudes, and poor practices about the prevention and control of NCC-induced epilepsy among HCWs in the study area. Most of the interviewed HCWs were not aware that NCC could cause epilepsy in humans, and more than half did not know that NCC in people with epilepsy could be treated. Further, the majority of HCWs didn’t know the diagnostic techniques and recommended drugs for NCC and NCC-induced epilepsy. Public health intervention programs aimed at preventing, controlling, and eradicating endemic poverty-related zoonotic diseases in many endemic areas should target both communities and HCWs in place. One Health approach is needed to promote better KAP among HCWs towards the prevention and control of acquired epilepsy in Tanzania.

## Introduction

Epilepsy is widely regarded as the most prevalent neurological disorder affecting people of all ages. An estimated 50 million people suffer from active convulsive epilepsy globally, with more than 85% of people with epilepsy (PWE) living in low-middle-income countries (LMICs) [1]. Sub-Saharan Africa (SSA) has approximately 9 million people with active epilepsy (PWAE) with higher prevalence in rural areas than in urban areas [2]. In Tanzania, knowledge of the burden of epilepsy is limited [3]. According to the World Health Organization (WHO) epilepsy report of 2022, many healthcare professionals in resource-limited countries lack knowledge in recognizing, diagnosing, treating, and controlling epilepsy and epileptic seizures.

One of the major causes of preventable epilepsy in LMICs is neurocysticercosis (NCC), a serious neurological zoonotic disease caused by the larval stage (cysticerci) of the pork tapeworm, *Taenia solium.* NCC has higher transmission in communities that practice *T. solium* risky behaviors [4], particularly in rural areas of SSA, Latin America, China, India subcontinent, South-East Asia, and Indian Ocean/Madagascar [5]. Further, different studies conducted elsewhere reported NCC cases in non-endemic countries due to an increased number of migrants from endemic *T. solium* areas [6–8]. Therefore, NCC is a global health challenge, and its transmission patterns could be projected to increase in non-endemic countries, causing substantial costs in the healthcare system. The transmission cycle of *T. solium* NCC is complex involving both humans and pigs. Humans harbor adult parasites in the small intestine and are capable of shedding *T. solium* eggs, which can infect both pigs and people. Pigs get infected by *T. solium* cysticerci by scavenging on human feces and eating feed or water contaminated by infective *T. solium* eggs [9]. In contrast, humans get infected through the fecal-oral route [10], autoinfection for carriers of adult *T. solium* tapeworms [11], and contaminated soil in both humans and pigs [12]. Currently, the diagnosis of NCC and NCC-induced epilepsy relies on neuroimaging techniques (computed tomography and magnetic resonance imaging) which are limited in LMICs [13], and unaffordable by low-income households [14].

Globally, NCC contributes to 30% of acquired epilepsy cases in endemic areas [13], and the association is estimated to up to 70% of epilepsy cases in high-risk communities [15]. NCC-induced epilepsy is a significant cause of disability and lifetime lost, impaired cognitive and poor quality of life [16], contributing to an increase in long-term health burdens on humans. Different neuroimaging studies conducted around the world have reported a substantial association between NCC and epilepsy with a study in Peru reporting 30% to 50% of NCC-induced epilepsy cases [17], about 61% of NCC in PWE in India [18], 80% of epilepsy cases had NCC in Madagascar [5], more than 50% of NCC in PWE reported in Mozambique [19] and a study in Zambia reporting about 60% [20]. The WHO’s 2021 NCC management guideline, estimated 2.56-8.30 million suffer symptomatic and asymptomatic NCC globally. Although the reported number of people suffering from NCC-induced epilepsy is high [4], data on prevalence and its burden in the SSA region is limited [2], despite being high endemicity of *T. solium* infections.

In Tanzania, Studies have indicated that NCC is prevalent among PWE and that its management is becoming remarkably more expensive, with estimated annual costs of about $ 5 million [14,21]. Further, a diagnosis of NCC-induced epilepsy costs between $140 and $180, which low-income households cannot afford [14]. In rural settings, stigmatization of PWE predominates, leading to traditional treatment methods [3]. Neuroimaging studies reported a prevalence ranging from 17.9% to 76% of PWE having NCC in the country [22–24]. These figures indicate the need for control of NCC-related epilepsy in Tanzania, which requires adequate knowledge of the parasite and its burden on communities by all stakeholders. Despite the endemic nature of *T. solium* NCC in rural communities, the KAP about the disease and its control among healthcare professionals in Tanzania is not well established. Additionally, the impact of NCC-related epilepsy on human health is less considered.Prevention and control of NCC-related epilepsy in endemic areas like Tanzania requires knowledgeable health professionals who are confronted with affected people. This study aimed to assess healthcare workers’ knowledge, attitudes, and practices (KAP) towards control and prevention of NCC-induced epilepsy in northern Tanzania.

## Materials and methods

### Ethics statement

Approval of this study was obtained from the National Health Research Ethics Committee (NatHREC) of the National Institute for Medical Research (NIMR) certificate number NIMR/HQ/R.8a/Vol.IX/4313 in Tanzania. Participation in the study by healthcare workers was voluntary and after a signed informed consent form. Also, respondents were assured that there were no risks to participating or not, and all information shared was coded in numbers to ensure confidentiality.

### Study design

A cross-sectional, healthcare professional-based KAP study was conducted between May and July 2023. A structured questionnaire was administered to assess healthcare professionals’ knowledge, practices, and attitudes regarding the prevention and control of NCC-induced epilepsy.

### Study area

This study was conducted in Mbulu and Babati rural districts in Manyara region (S1 Fig), which are known to be endemic for *T. solium* infections, with a reported porcine cysticercosis (PCC) and human cysticercosis (HCC)/NCC prevalence of at least 12% and greater than 16%, respectively, in northern Tanzania [21]. Mbulu District is the most known hyperendemic epidemiological area for porcine cysticercosis and human cysticercosis/NCC in northern Tanzania [23,25]. It has a population of 376,865 people [26], and 35wards with 45 primary health facilities registered to provide health services to the community. Based on the study done by [27], most pig farmers practice the free-range system.

In Babati district, pig-keeping is one of the main activities carried out in the area as a source of household income and animal protein. Free-range keeping of pigs dominates after crop harvest periods. An epidemiological study reported a prevalence of 13% porcine cysticercosis based on lingual examination [28], indicating that *T. solium* cysticercosis is endemic in the area. The study area (both districts) has a total number of 60 wards and 103 primary healthcare facilities located in various wards.

The study was conducted in only two districts namely Mbulu and Babati rural, and focused on assessing the knowledge, attitudes, and practices of healthcare workers (HCWs) in the primary health facilities (namely dispensaries, health centers, and district hospitals), towards control and prevention of NCC-related epilepsy in the Manyara region of northern Tanzania. People with epilepsy (PWE) and community members were not included in the study.

### Study population and sampling frame

Healthcare services in Tanzania are decentralized locally into dispensaries, health centers, and district hospitals forming the primary healthcare system. The target population in this study were primary healthcare professionals, and they were categorized into sub-groups of assistant physicians (clinical assistants, clinical officers, assistant medical officers), physicians (medical doctors), health laboratory technologists, community health workers, and nurses working in the primary healthcare facilities (PHFs) within the study area. A sampling frame of HCWs was obtained from the offices of District Medical Officers (DMO) in the two districts.

### Sample size and sampling methods

The targeted sample size was 250 respondents and was calculated using the formula developed by Israel, (1992)**: n = no/{1+(no-1/N**)}, where (no = Z²pq/d²) at a 95% confidence interval and **N** is the total number of healthcare workers in the study districts.

From the formula; (no = Z²pq/d²); **no** is the sample size, **Z** is the confidence coefficient when α = 0.05, **d** is the absolute precision, and **p** is the estimated proportion of the study subjects, whereas **q** is equal to 1-p. Since the estimated proportion (p) of the outcome variables (knowledge, attitudes, and practices) among healthcare professionals in Babati rural and Mbulu districts was unknown, then 50% of the proportion (P) estimates was assumed to calculate the sample size. When d = 0.05, z = 1.96, p = 0.5, and q = 0.5 are substituted, then no = 385.


$$\text{Sample\textbackslash{} size }n_{0}=\frac{z^{2}pq}{d^{2}}\;\left(\frac{1.96^{2}\times 0\cdot 5\times 0\cdot 5}{0.05^{2}}\right)=384.16=385$$

According to the records obtained from the office of the DMO from Mbulu and Babati rural districts, the total number of healthcare providers (N) was 710, and the number in subgroups was 404 nurses, 48 health laboratory technologists, 86 community health workers, 43 physicians/medical doctors, and 129 assistant physicians (including 28 clinical assistants, 83 clinical officers, and 18 assistant medical officers). Therefore, the adjusted sample size (n) of the study respondents (healthcare workers) was obtained from the formula below [29,30].


$$\text{Adjusted\textbackslash{} sample\textbackslash{} size }(n)=\frac{n_{0}}{1+\left(\frac{n_{0}-1}{\text{N}}\right)}=\frac{385}{1+\left(\frac{385-1}{710}\right)}=249.86=250\text{ healthcare\textbackslash{} workers}$$

The number of healthcare workers by categories was obtained using the proportion-to-size sampling techniques, as given by the formula below;


$$\begin{array}{c} \text{ Professional\textbackslash{} category}=\frac{Np}{N}\times n\text{ as\textbackslash{} adapted\textbackslash{} from} \end{array}$$

[30]

Whereby; **Np** = Number of professional categories in the study area, **N**= (710) Total number of healthcare workers in the study area, and **n**=(250) Adjusted sample size of the study. Therefore, the number of healthcare workers who participated in the study were 17 health laboratory technologists, 142 Nurses, 30 community health workers(CHWs), 16 Physicians (medical doctors), and 45 assistant physicians

Participants were randomly selected with equal sampling fractions of 50% in the two districts. A simple random sampling technique was used to select the number of health facilities using random numbers representing primary health facilities (PHFs). A total of 32 PHFs were randomly selected out of 103. At each primary health facility, a sampling fraction of 40% was used to recruit respondents. Further, HCWs were randomly selected from each subgroup or professional cadres using a stratified random sampling approach.

### Participants recruitment procedures

Primary healthcare facility with at least four HCWs was included in the study, meanwhile, HCWs with at least one year of working at a PHF were included in the study. Secondary and tertiary healthcare facilities were not included in the study. The study protocol was well introduced, and respondents were allowed to participate in the study after signing the informed consent form.

### Data collection tools and procedures

A structured questionnaire with closed-ended questions in English language (S1 Appendix) was developed through an in-depth literature search and administered face-to-face with the study participants (healthcare workers) to collect information about their socio-demographic characteristics and to assess their knowledge, attitudes, and practices about NCC-related epilepsy. The internal consistency and reliability of the questionnaire were pre-validated, and the Cronbach‘s alpha coefficients for knowledge, attitudes and practices were adopted from an existing study [31]

The questionnaire had 51 questions and four parts, including socio-demographic characteristics (part I) with a set of five questions. Part II (knowledge section) had a set of fifteen questions including seven questions with multiple responses. Part III (attitudes section) had a set of ten questions including three questions with multiple responses, and part IV (practices section) had twenty-six questions including six questions with multiple responses. All questions had two possible answers (YES/NO), and in the case of questions with multiple responses, each response was considered independently.

Further, the questionnaire was pre-tested through a pilot study (in a different district) for readability, comprehensibility, relevance, clarity, and accuracy. Piloted questionnaires were administered to 23 participants based on the sub-groups: 5 nurses, 5 health laboratory technologists, 5 assistant physicians, 5 physicians, and 3 community health workers, all of whom were interviewed but were excluded from the final data analysis. Some questions regarding the knowledge section were rephrased for understandability after pre-testing. The questionnaire translated into Kiswahili language was used to collect information from community health workers (CHWs) and nurses who opted to use it.

### Establishing KAP scores

The KAP variables were assessed to obtain the respondents’ performance scores on NCC-induced epilepsy. Administered questions were answered based on “YES or NO” where a correct response from the respondent was given 1 point and an incorrect or I don’t know the response was given 0 points [31].

The overall KAP scores were assigned from 0–100%, and the mean KAP scores of the respondents were calculated from KAP-tested variables. The percentage of individual KAP scores in the study was calculated to obtain a continuous variable for comparing the mean scores of respondents, and likewise to obtain the dichotomous outcomes for the level of respondents’ KAP. The percentage scores out of the maximum score cut-point of 70%, 75%, and 70% for knowledge, attitudes, and practices, respectively, were used to determine the performance scores, as adapted from [32,33].

Therefore, respondents with performance scores of ≥70%, ≥ 75%, and ≥70% were deemed to have adequate knowledge, positive attitudes, and good practices, respectively, regarding the control and prevention of NCC-induced epilepsy. Likewise, respondents with performance scores ranging from 0–69%, 0–74%, and 0–69% were considered to have inadequate knowledge, negative attitudes, and poor practices, respectively, towards the prevention and control of NCC-induced epilepsy.

### Data analysis

#### Data acquisition.

Quantitative data for analysis was generated after administering a structured questionnaire to healthcare workers to capture socio-demographic factors and their knowledge, attitudes, and practices toward NCC-induced epilepsy. The gathered information was entered and coded into numbers in the Microsoft Excel Spreadsheet 2019, creating a datasets (S1 Dataset). All datasets were cleaned, validated, and finally uploaded into Statistical Package for Social Science (SPSS statistics, version 26) for univariate analysis, bivariate analysis, and multivariate analysis.

#### Statistical analysis of the generated data.

Analytical and descriptive statistics were performed to generate the results. In this study, the outcome variables (KAP scores of the respondents towards NCC-induced epilepsy), and independent variables (study districts, sex, age, educational levels, professional category, and working experience) were identified. Univariate analysis was used to produce the frequency and proportions of the measured variables.

Knowledge, attitudes, and practices scores generated from the individual percentage scores were identified as continuous variables. An independent two-sampled T-test was conducted to compare the mean scores of continuous variables against the independent factors with two possible groups; study districts (Mbulu and Babati rural), and sex (males and females). Significant statistics were considered at *p-value<*0.050*,* with equal variances assumed across all groups. Analysis of variance (One-way ANOVA, Post Hoc tests using the Tukey method) was run to compare the mean scores and/or mean differences of KAP scores between the respondents’ professional category, educational levels, age group, and working experience towards NCC-associated epilepsy. The Levene test was used to check the rule of homogeneity that was validated at *p-value* > 0.050, and the significant statistics for the mean KAP scores between comparable groups were considered at *p* < 0.050.

Analysis of factors associated with KAP scores was conducted using bivariate analysis and a step-wise binary logistic regression model. In the bivariate analysis, for categorical variables with dichotomous outcomes (the level of KAP performance), Pearson chi-square was fitted to test the association of independent variables, and the significant association was considered at p-value<0.050. A Linear regression model was run to assess the association of other independent factors, and the percentage of individual KAP scores was used as an outcome variable. The independent variables retained significance at a cut-point of p ≤ 0.20 and were considered for the next step [32].

A Step-wise binary logistic regression model (Forward-Likelihood Ratio Method), was used to test the strength of associations and/or the predictors of outcomes of interest, whereas outcome variables (KAP scores) with binary outcomes fitted the model, and the statistical significance at 95% confidence interval was considered at p-value < 0.050, and odds ratio (OR) > 1 for a good predictor, and OR < 1 was considered as a protective factor. The fitted statistics were validated using the Omnibus tests of model coefficients, where the coefficients (factors) were considered statistically significant in the model at p < 0.050, and the goodness of fit of the model was tested using the Hosmer-Lemeshow test, and the model was fitted at an insignificance p > 0.05.

## Results

### Socio-demographic characteristics of the study population

A total of 250 respondents (healthcare workers) participated in this study at an equal ratio of fifty percent (125/250) from Babati rural and Mbulu districts (Table 1). Male participants were 50.8% while females were 49.2%, with ages ranging from 20 to 56 years. Based on the professional category of respondents, 56.8% were nurses, 12% were community health workers, 6.8% were health laboratory technologists, 18% were assistant physicians, and 6.4% were physicians/medical doctors. The majority of participants (80%) attained middle college-level education (certificates, diploma, advanced diploma), while 11.2% attained basic level education (primary and secondary), and only 8.8% obtained tertiary level education (bachelor’s and master of science degrees).

**Table 1:** Socio-demographic characteristics of the study participants.

Variable	Category	Frequency (n = 250)	Proportion %
Study area (districts)	Mbulu	125	50
	Babati rural	125	50
Gender (sex)	Females	123	49.2
	Males	127	50.8
Age (years)	20-29	73	29.2
	30-39	95	38.0
	40-49	49	19.6
	50-59	33	13.2
Educational level	Basic education	28	11.2
	Middle College Education	200	80.0
	Tertiary education	22	8.8
Professional category	Health laboratory technologists	17	6.8
	Community health workers	30	12.0
	Nurses	142	56.8
	Assistant physicians	45	18.0
	Physicians (medical doctors)	16	6.4
Working experience	1-5 years	113	45.2
	6-10 years	62	24.8
	11-15 years	39	15.6
	16-20 years	17	6.8
	Above 20 years	19	7.6

### Knowledge of neurocysticercosis-induced epilepsy

#### Participants knowledge scores.

Participants’ mean knowledge scores in this study were 55.5% (Table 2), which indicated inadequate knowledge (at a cut-point of 70%). The majority of the participants (81.2%) reported knowing about NCC. However, despite more than half (54.4%) knowing its zoonotic implications, only 25.2% knew how humans acquired the infection of NCC. The relationship between NCC and epilepsy is still underrated, as only 49.2% of the respondents were aware of the relationship, and only 45.6% of the respondents knew that NCC in PWE could be treated.

**Table 2:** Knowledge scores of the participants towards NCC-induced epilepsy.

Tested Knowledge variables	Response	Frequency (n = 250)	Proportion %	Knowledge scores
Do you know Neurocysticercosis (NCC)	YES NO	203 47	81.2** 18.8	81.2
Is it a zoonotic disease?	YES NO	136 114	54.4** 45.6	54.4
Do you know the cause of Neurocysticercosis	YES NO	179 71	71.6** 28.4	71.6
Hosts involved in the transmission cycle of NCC	YES NO	129 121	51.6** 48.4	51.6
How people acquire the infection of NCC	YES NO	63 187	25.2** 74.8	25.2
Relationship between NCC and epilepsy	YES NO	123 127	49.2** 50.8	49.2
Common symptoms in NCC patients	YES NO	139 111	55.6** 44.4	55.6
Do you know that NCC can cause Epilepsy?	YES NO	128 122	51.2** 48.8	51.2
NCC in people with epilepsy (PWE) can be treated?	YES NO	114 136	45.6** 54.4	45.6
Knowledge of the prevention and control measures of NCC-induced epilepsy	YES NO	173 77	69.2** 30.8	69.2
Participants’ average knowledge scores %			(554.8/10)	55.5

n = Number of participants; ** = Percentage for knowledge scores

#### Participants mean knowledge scores across all the independent variables.

Variations of knowledge scores among respondents were observed through a comparison of mean scores and/or mean differences. The mean score for knowledge in Mbulu district was statistically higher (p < 0.01) than in the Babati rural district. A high level of education was significantly associated with better knowledge of NCC-induced epilepsy among HCWs (p* *< 0.01). Based on the professional categories of respondents, the mean knowledge scores were statistically higher (70.94 ± 13.645; p < 0.01) in health laboratory technologists compared to nurses and community health workers (Table 3).

**Table 3:** Mean KAP scores.

Variable	Frequency (N = 250)	Knowledge scores			Attitude scores			Practices scores		
		Mean	Std dev	95% CI	Mean	Std dev	95% CI	Mean	Std dev	95% CI
**Study districts**										
Mbulu	125	58.10	19.437	54.66-61.54	56.82	14.876	54.19-59.46	48.76	17.363	45.69-51.83
Babati rural	125	51.05	20.602	47.40-54.70	50.90	14.430	48.25-53.46	48.93	15.583	46.17-51.69
		P = 0.006**			P = 0.002**			***P = 0.936***		
**Sex**										
Females	123	53.23	19.701	49.71-56.74	53.35	14.917	50.69- 56.01	47.70	15.776	44.88-50.52
Males	127	55.87	20.853	52.21-59.54	54.36	14.971	51.73- 56.99	49.95	17.094	46.95-52.95
		***P = 0.304***			***P = 0.593***			***P = 0.280***		
**Age**										
20-29 years	73	54.37	19.455	49.83-58.91	48.88	15.702	45.21-52.54	47.36	16.562	43.49-51.22
30-39 years	95	52.71	21.929	48.24-57.17	54.83	14.378	51.90-57.76	48.60	16.701	45.20-52.00
40-49 years	49	53.33	20.319	47.49-59.16	56.06	14.405	51.92-60.20	50.35	15.463	45.91-54.79
50-59 years	33	62.24	15.770	56.65-67.83	58.85	12.923	54.27-63.43	50.61	17.385	44.44-56.77
		***P = 0.126***			P = 0.004**			***P = 0.707***		
**Education levels**										
Basic education	28	52.79	16.824	46.26-59.31	49.39	15.149	43.52-55.27	41.64	12.458	36.81-46.47
Middle College Education	200	53.65	20.813	50.75-56.55	53.12	14.757	51.06-55.18	48.77	16.901	46.41-51.12
Tertiary education	22	65.23	16.873	57.75-72.71	66.32	9.357	62.17-70.47	58.73	11.679	53.55-63.91
		P = 0.035**			P = 0.000**			P = 0.001**		
**Professional category**										
Community health workers	30	51.30	16.242	45.24-57.36	47.80	14.826	42.26-53.34	41.10	12.254	36.52-45.68
Nurses	142	50.10	21.139	46.59-53.61	51.93	15.037	49.43-54.42	49.28	16.593	46.53-52.03
Health Laboratory technologists	17	70.94	13.645	63.93-77.96	60.06	12.867	53.44-66.67	42.88	18.871	33.18-52.58
Assistant physicians	45	61.49	16.796	56.44-66.54	58.00	13.706	53.88-62.12	51.29	16.380	46.37-56.21
Physicians (doctors)	16	63.56	18.910	53.49-73.64	64.19	10.061	58.83-69.55	58.94	12.887	52.07-65.80
		P = 0.000**			P = 0.000**			P = 0.003**		
**Working experience**										
1-5 years	113	52.30	20.867	48.41-56.19	50.61	14.576	47.89-53.33	48.26	17.381	45.02-51.50
6-10 years	62	55.06	18.614	50.34-59.79	53.76	15.693	49.77-57.74	48.53	16.230	44.41-52.65
11-15 years	39	58.10	22.599	50.78-65.43	59.05	14.542	54.34-63.77	50.00	15.209	45.07-54.93
16-20 years	17	53.59	21.849	42.35-64.82	56.82	15.444	48.71-64.76	46.88	16.412	38.44-55.32
Above 20 years	19	60.11	14.806	52.97-67.24	60.26	9.451	55.71-64.82	52.74	14.996	45.51-59.96
		***P = 0.397***			P = 0.006**			***P = 0.800***		

** = Indicates significance statistics at *p-value* < 0.05; Std dev = Standard deviation; CI = Confidence interval at α = 0.05

#### Predictors of knowledge scores.

Professional categories and study districts were determined as good predictors of knowledge scores for NCC-induced epilepsy (Table 4). Respondents from Mbulu district were 1.926 times more knowledgeable about controlling and preventing NCC-induced epilepsy (OR: 1.926, 95% CI: 1.046–3.545; p < 0.05) compared to those from Babati district. Health laboratory technologists were observed to be more knowledgeable (OR: 5.821, 95% CI: 2.033–16.664, p < 0.01) about NCC-induced epilepsy, followed by assistant physicians (OR: 3.141, 95% CI: 1.484-6.650, p < 0.01) compared to nurses.

**Table 4:** Predictors of knowledge scores.

Predictor variable	Frequency (n = 250)	OR	95% CI	*P-value*
**Study districts** (n = 250)				
Mbulu district	125	1.926	1.046-3.545	0.035**
Babati rural district	125	Ref		
**Professional category** (n = 250)				
Nurses	142	Ref		
Health laboratory technologists	17	5.821	2.033- 16.664	0.001**
Assistant physicians	45	3.141	1.484- 6.650	0.003**
Physicians/medical doctors	16	1.725	0.511- 5.821	0.380
Community health workers	30	0.575	0.161- 2.054	0.394

Ref = reference category; ** = Significant *p-*value; OR: Odds Ratio; CI = Confidence interval at α = 0.05

### Attitudes towards neurocysticercosis-induced epilepsy

#### Participants attitude scores.

Participants’ mean attitude scores were 54.0%, which indicated negative attitudes (at a cut point of 75%) towards control and prevention of NCC-induced epilepsy. Of the respondents, (163/250) believed that epilepsy is inherited from parents, and 38.4% did not believe in modern treatment (modern medicine) for PWE. Furthermore, we found that 46.8% of the respondents reported that the pork tapeworm *T. solium* could cause human epilepsy (Table 5).

**Table 5:** Participants’ attitude scores towards NCC-induced epilepsy.

Tested variables	Response	Frequency (n = 250)	Proportion%	Attitude scores
Have you heard of a disease called epilepsy	YES NO	243 12	95.2 4.8	
Believe that Epilepsy is an inherited disease or disorder from parents or ancestors	Incorrect Correct	163 87	65.2 34.8**	34.8
The trend of epilepsy cases at the health facility				
Increasing	YES NO	65 185	26.0** 74.0	26.0
Decreasing	YES NO	130 120	52.0** 48.0	52.0
Do you believe that the tapeworm *Taenia solium* can cause epilepsy?	YES NO	117 133	46.8** 53.2	46.8
Believe that people who eat raw or undercooked pork are more likely to acquire epilepsy	YES NO	219 31	87.6** 12.4	87.6
Believe that unhygienic use of toilets is a risk factor for acquiring NCC-induced epilepsy	YES NO	113 137	45.2** 54.8	45.2
Whether people with epilepsy can be diagnosed	YES NO	152 98	60.8** 39.2	60.8
Whether people with epilepsy can be treated	YES NO	179 71	71.6** 28.4	71.6
Believes in the modern treatment to be provided to people with epilepsy (PWE)	YES NO	154 96	61.6** 38.4	61.6
Participants’ average attitude scores %			(486.4/9)	54.0

** = Percentage for attitude scores; n = Number of participants

#### Participants mean attitude scores across all the independent variables.

Respondents from Mbulu district were associated with positive attitudes towards NCC-induced epilepsy than respondents from the Babati rural district p < 0.01 (Table 3). Further, increasing the age of respondents was statistically significantly associated with better attitudes towards control and prevention of NCC-induced epilepsy (p < 0.01). Based on the professional category of the respondents, the mean attitude scores were statistically higher (64.19 ± 10.061; p < 0.01) in medical doctors compared to nurses and community health workers. Respondents who attained tertiary education were significantly associated with better attitudes toward NCC-induced epilepsy compared to respondents who attained other education levels (p < 0.01). A greater number of years of working experience was associated with better attitudes toward the control and prevention of NCC-induced epilepsy among healthcare workers.

#### Predictors of attitude scores.

Study districts, educational levels, and knowledge levels were identified as significant predictors of a positive attitude toward the control and prevention of NCC-induced epilepsy (Table 6). Respondents from Mbulu district were three (OR: 3.228, 95% CI: 1.297–8.037, p < 0.05) times more likely to have a positive attitude towards preventing and controlling a disease compared to respondents from Babati rural district*.* Meanwhile, respondents with a tertiary level of education were three (OR: 3.361; 95% CI: 1.147–9.848, p < 0.05) times more likely to have a positive attitude towards preventing and controlling NCC-induced epilepsy compared to respondents with middle college educational levels.

**Table 6:** Predictors of attitude scores.

Predictor variables	Frequency (n = 250)	OR	95% CI	*P-value*
**Study districts** (n = 250)				
Mbulu district	125	3.228	1.297-8.037	0.012**
Babati rural district	125	Ref		
**Educational levels** (n = 250)				
Basic education	28	0.325	0.041- 2.552	0.285
Tertiary education	22	3.361	1.147- 9.848	0.027**
Middle College Education	200	Ref		
**Knowledge levels (n = 250)**				
Inadequate knowledge	194	Ref		
Adequate knowledge	56	6.655	2.872-15.421	0.000**

Ref: Reference category; OR: Odds Ration; **: Indicates Significant p-value

### Practices towards neurocysticercosis-induced epilepsy

#### Participants practice scores.

In this study, we found that the average practice scores of the participants were 49.3%, which indicated poor practices (at a cut-point of 70%). Of the respondents, 51.2% could diagnose NCC-induced epilepsy, and only 20.1% knew the diagnostic techniques to use. Further, 33.8% knew the recommended drugs for the treatment of NCC-induced epilepsy, however, half of the respondents (50%) relied on anti-seizure drugs to manage PWE (Tables 7 and 8).

**Table 7:** Participants’ practice scores towards NCC-induced epilepsy; diagnosis practices.

Tested variables	Response	Frequency (n = 250)	Proportion %	Practice scores %
Have you met with people with epilepsy (PWE)	YES NO	238 12	95.2** 4.8	95.2
Do you manage PWE at your health facility	YES NO	215 35	86.0** 14.0	86.0
Can you diagnose epilepsy?	YES NO	223 27	89.2** 10.8	89.2
Where is the diagnosis done?				
At dispensaries	YES NO	154 96	61.6** 38.4	61.6
At the health centers	YES NO	181 69	72.4** 27.6	72.4
At the hospital	YES NO	188 62	75.2** 24.8	75.2
Do you consider NCC as part of differential diagnosis for epilepsy cases	YES NO	148 102	59.2** 40.8	59.2
Do you diagnose NCC-induced epilepsy?	YES NO	128 122	51.2** 48.8	51.2
What diagnostic techniques do you use?				
Magnetic resonance imaging (MRI)	YES NO	52 198	20.8** 79.2	20.8
Computed tomography (CT) scanning	YES NO	66 184	26.4** 73.6	26.4
Immunodiagnostic techniques (ELISA or EITB)	YES NO	33 217	13.2** 86.8	13.2
The neuroimaging technique is ideal for diagnosis of NCC in PWE)	YES NO	128 122	51.2** 48.8	51.2
Use of a standard chart of diagnostic criteria for NCC in PWE which is widely used worldwide?	YES NO	50 200	20.0** 80.0	20.0
Do you provide a referral for PWE to higher hospitals for further diagnosis and treatments?	YES NO	146 104	58.4** 41.6	58.4

**Table 8:** Participants’ practice scores towards NCC-induced epilepsy; treatment and case follow-up.

Tested variables	Response	Frequency (n = 250)	Proportion%	Practice scores %
Practice after diagnosed epilepsy case at the health facility				
Treat	YES NO	179 71	71.6** 28.4	71.6
Refer to a higher hospital	YES NO	168 82	67.2** 32.8	67.2
NCC-induced epilepsy can be treated?	YES NO	135 115	54.0** 46.0	54.0
Use the national guideline or WHO guideline for the management of people with epilepsy/epileptic seizures due to NCC	YES NO	167 83	66.8** 33.2	66.8
Recommended drugs for the treatment of NCC in PWE.				
Praziquantel	YES NO	90 160	36.0** 64.0	36.0
Albendazole	YES NO	111 139	44.4** 55.6	44.4
Combination of Albendazole and Praziquantel	YES NO	92 158	36.8** 63.2	36.8
Anti-seizures	YES NO	125 125	50.0** 50.0	50.0
Anti-inflammatories such as dexamethasone	YES NO	57 193	22.8** 77.2	22.8
Analgesics	YES NO	76 174	29.6** 70.4	29.6
Anti-edema such as mannitol	YES NO	42 208	16.8** 83.2	16.8
Do you titrate the medication for your epilepsy patients?	YES NO	143 107	57.2** 42.8	57.2
Active follow-ups for patients on treatment with anti-epileptic drugs (AEDs)	YES NO	191 59	76.4** 23.6	76.4
Do patients who are on AEDs report back to your facility?	YES NO	199 51	79.6** 20.4	79.6
Do you know of any epilepsy patients that have come off treatment?	YES NO	108 142	43.2** 56.8	43.2
If yes, to the question above, what reasons do they give for stopping treatment?				
Side effects	YES NO	62 188	24.8** 75.2	24.8
Treatment not working	YES NO	45 205	18.0** 82.0	18.0
Run out of drugs	YES NO	57 193	22.8** 77.2	22.8
What do you do for patients who have stopped treatment?				
Change the drug	YES NO	99 151	39.6** 60.4	39.6
Change the dosage of the drug they are on	YES NO	83 167	33.2** 66.8	33.2
Refers to higher hospitals	YES NO	129 121	51.6** 48.4	51.6
Do you report a suspected case of NCC-induced epilepsy	YES NO	132 118	52.8** 47.2	52.8
Average practice scores of the participants (%)		Table (7 + 8)	(1,775.2/36)	49.3

** = Percentage for practices scores; n = Number of participants

#### Participants mean practice scores across all the independent variables.

A high level of education was significantly associated with better practices (*p* < 0.01) among respondents towards control and prevention of NCC-induced epilepsy. Based on the professional category of respondents, the mean practice scores were statistically higher (58.94 ± 12.887; *p* < 0.01) in medical doctors compared to health laboratory technologists and community health workers (Table 3)

#### Predictors of practice scores.

There was no significant association between the independent factors (sex, age, education level, working experience, professional categories, study districts) and practice scores (p > 0.05), suggesting that there were no predictors of practice management among all respondents in the study area.

### Respondents KAP performance scores toward Neurocysticercosis-induced epilepsy

The level of knowledge, attitudes, and practices among respondents was generated from individual percentage scores at cut-points of 70%, 75%, and 70% respectively, as the dichotomous outcome variables. Of the respondents, only 22.4%, 10.8%, and 12.8% had adequate knowledge, positive attitudes, and good practices towards NCC-induced epilepsy, respectively (Table 9).

**Table 9:** The level of KAP performance among HCWs towards NCC-induced epilepsy.

Outcome Variable	Frequency (n = 250)	Cut-point	Proportion %	95% CI
**Knowledge**				
Adequate knowledge	56	≥70%	**22.4%**	44-70
Inadequate knowledge	194	≤69%	77.6%	180-206
**Attitudes**				
Positive attitudes	27	≥75%	**10.8%**	19-38
Negative attitudes	223	≤74%	89.2%	212-231
**Practices**				
Good practices	32	≥70%	**12.8%**	23-43
Poor practices	218	≤69%	87.2%	207-227

### Association between knowledge, attitudes, and practices toward NCC-induced epilepsy

The level of knowledge was identified as an outcome variable with scores of ≥70% for good knowledge and <70% for low knowledge, whereas practices and attitude scores were identified as predictor variables. Pearson chi-square was conducted to determine the association between good knowledge, good practices, and positive attitudes. There was a statistically significant association between good knowledge and good practices ($\chi^{2}$ = 19.930; p < 0.01) and good knowledge and positive attitudes ($\chi^{2}$ = 23.658; p < 0.01)

A step-wise binary logistic regression model was run to determine the strength of associations between good knowledge, good practices, and positive attitudes. We found that HCWs with good practices were four times, and positive attitudes were six times, more likely to have good knowledge of the prevention and control of NCC-induced epilepsy in the study area (p < 0.01) (Table 10).

**Table 10:** Association between the level of knowledge, attitudes, and practices.

Factor	Frequency (n = 250)	Cut-point	OR	95% CI	*P-value*
**Attitudes scores** (n = 250)					
Positive attitudes	27 (10.8%)	≥75%	6.087	2.534-14.622	0.000
Negative attitudes	223 (89.2%)	≤74%	Ref		
**Practice scores** (n = 250)					
Good practices	32 (12.8%)	≥70%	4.750	2.099-10.748	0.000
Poor practices	218 (87.2%)	≤69%	Ref		

Ref: Reference category; OR: Odds Ration; n = Total number of respondents

## Discussion

This is the first healthcare professional-based KAP study towards control and prevention of NCC-induced epilepsy conducted in *T. solium* endemic areas in Tanzania. This study revealed that healthcare workers (HCWs) had overall inadequate knowledge, negative attitudes, and poor practices towards control and prevention of NCC-induced epilepsy in Tanzania.

### Knowledge towards control and prevention of NCC-induced epilepsy

Our study established that more than three-quarters of HCWs had insufficient knowledge of NCC-induced epilepsy. The knowledge of HCWs regarding the transmission cycle of NCC and treatment of NCC in PWE was low, and the majority of the HCWs did not know how humans get infected with NCC. This finding highlights inadequate continuing medical education programs in primary healthcare facilities. A targeted training programs among HCWs, focused on neglected zoonotic diseases like NCC is crucial for the effective prevention and control of NCC-induced epilepsy and other endemic neglected zoonotic diseases. Knowledge of the transmission cycle, risk factors, and prevention methods of NCC-induced epilepsy could empower HCWs to educate communities, ultimately leading to a reduction in the incidence of acquired epilepsy cases in the study area.

Previous studies not specific to KAP-based have reported similar findings as the present study. A study in Mbeya Tanzania, revealed insufficient knowledge among health professionals on the infection of NCC [34]. Another study conducted in the endemic regions for NCC in Tanzania, reported that more than half (57.9%) of the healthcare providers in the primary healthcare facilities had poor knowledge of *T. solium* cysticerci infections [9].

Furthermore, we found that 18.8% of HCWs were not aware of NCC despite the endemic nature of *T.solium* tapeworm in the study area. This indicated that a lack of awareness of NCC among HCWs could impact both the diagnosis and treatment options for PWE due to NCC, and possibly limit public health interventions for NCC-induced epilepsy in rural communities. This figure is lower than previously reported in Eastern Zambia [35] and in Bolivia [36]. In Zambia, approximately 42% of healthcare workers didn’t know about NCC [35]. Meanwhile, 90% of respondents were not aware of NCC and had limited knowledge about how humans get infected with *T. solium* cysticerci in Bolivia [36]. KAP studies, healthcare professional-based regarding NCC and epilepsy are limited in endemic regions. Therefore, the findings of this study would contribute to the gaps in knowledge, attitudes, and health practices associated with NCC-induced epilepsy in *T. solium* endemic settings, using quantitative research methods.

The present study also found that HCWs had limited knowledge about the association between NCC and epilepsy with 51% not aware of the relationship. Most of the interviewed HCWs (48.8%) were not aware that NCC could cause epilepsy in humans, and more than half did not know that NCC in PWE could be treated. This indicated that most of HCWs in primary healthcare facilities are not fully aware of the clinical presentation of NCC-induced epilepsy, and acquired epilepsy in general. However, the level of understanding among the HCWs could have been influenced by their scopes and responsibilities within their healthcare facilities. The transmission cycle of *T. solium* establishes a complex network between humans and pigs, causing great challenges in suspecting, diagnosing, and treating the parasite.

A better knowledge of healthcare professionals and the community is required for the control and eradication of this parasite in endemic areas. Our findings align with other studies that are not KAP-related, conducted elsewhere. More than half (56%) of healthcare providers in Zambia were not aware of the link between NCC and epilepsy [35]. Also, some previous studies that used a qualitative approach reported similar findings to our study results in Uganda [37] and in Bolivia [36].

Additionally, the professional category was associated with the level of knowledge of the HCWs in the study area. We observed that health laboratory technologists were five times more knowledgeable, followed by assistant physicians about NCC-induced epilepsy compared to other professional groups.

In the study area, assistant physicians are more prevalent in primary healthcare facilities, which may lead them to encounter PWE more frequently in their daily practice. This increased exposure could enhance their understanding and awareness of NCC-induced epilepsy and possibly other conditions. Observed high level of knowledge among health laboratory technologists could have been influenced by their substantial exposure to the diagnosis of *T. solium* taeniasis due to the endemicity of the tapeworm in the study area [23,25,28].

Related studies conducted elsewhere revealed significant differences in knowledge levels among healthcare professionals. Clinical officers had a higher level of knowledge compared to nurses (p < 0.050) about epilepsy in Zambia [38], whereas a study in Indian districts reported a signifiant difference in epilepsy knowledge levels among medical officers, nurses, and accredited social health activists (community health workers) at p < 0.050 [39]. Comprehensive understandings and different levels of knowledge among primary HCWs could be influenced by different levels of education, training coverage, scopes, and their responsibilities of delivering healthcare services to the communities [39].

### Attitudes towards control and prevention of NCC-induced epilepsy

This study found that the majority of HCWs had negative attitudes towards NCC-induced epilepsy. Attitudes influence the process of altering individuals’ perceptions, beliefs, thinking, or thoughts, and negative attitudes may lead to negative outcomes and vice-versa [31,40]. In the present study, 38.4% of HCWs did not believe in the use of modern medicine for the treatment of PWE, 39.2% believed that epilepsy could not be diagnosed, and 28.4% believed that epilepsy could not be treated. Negative beliefs among HCWs about the effectiveness of modern drugs, such as antiparasitic, ant-inflammatory, anti-edema, and antiepileptic medications, could hinder proper care for PWE with NCC, impacting referrals for diagnosis and treatment and potentially leading to a reliance on traditional treatment options. Additionally, negative attitudes and/or perceptions of HCWs in primary health facilities may disappoint the number of PWE from obtaining biomedical treatments [39], and may increase treatment gaps for PWE in Tanzania. Similarly, these findings were reported elsewhere in Burkina Faso by a study that utilized a qualitative research approach [41]. A general study done elsewhere in India reported that about 27.5% of medical officers believed in the use of traditional medicine for the treatment of epilepsy [39].

Further, the study found that 4.8% of HCWs had never heard about epilepsy. Surprisingly 65.2% of HCWs believed that epilepsy is a hereditary disorder or disease. These findings highlight significant gaps in awareness and attitudes, which could ultimately affect the management of PWE in primary healthcare settings. The primary HCWs are confronted first with PWE, therefore, options for diagnoses, treatments, and control of the disease could be affected by their perceptions and/or beliefs. In addition, according to the District Health Information System (DHIS) 2022, a total of 2,488 epilepsy cases were reported in the study area, with 1,166 cases in Mbulu and 1,322 cases in Babati rural. However, these figures were not specified whether the cases are acquired or inherited. These findings call for urgent national health interventions to advocate for and create health awareness among HCWs in primary health facilities regarding acquired epilepsy cases, including NCC-induced epilepsy, across the country.

Different from our study; about (9.2%) of nurses and (23.6%) of accredited social health activists had never heard about epilepsy in India [39], this finding is higher compared to that reported by the present study. Also, a study in Zambia reported that more than half of the primary healthcare providers classified epilepsy as a mental health ailment [38], whereas another study found that healthcare workers in rural settings in Burkina Faso, believed that most cases of epilepsy are associated with evil spirits [41]. However, this study was conducted using a qualitative approach, while our study employed quantitative methods. Negative attitudes or perceptions, and limited knowledge among primary HCWs may contribute to a substantial burden of epilepsy in the rural communities.

### Practices regarding control and prevention of NCC-induced epilepsy

The study found that the majority of HCWs had poor practices in managing PWE, although (86%) managed PWE at their health facilities. This showed that a large number of PWEs come from rural communities where they normally access health services from primary healthcare facilities. The present study identified large gaps in practice management among HCWs regarding their practices in diagnoses and drug prescriptions for the treatment of NCC-induced epilepsy.

The majority of HCWs didn’t know the diagnostic techniques for NCC and NCC-induced epilepsy, with 80% not aware of the use of a standard chart of diagnostic criteria for NCC in PWE, which is recommended by WHO worldwide [42]. In endemic *T. solium*, this could lead to misdiagnoses or underdiagnoses of NCC-induced epilepsy. This indicated that diagnosis of PWE in rural communities is limited and possibly hampered by inadequate knowledge, low diagnostic capacity, and negative attitudes among primary HCWs in the area. This could be the reason that acquired epilepsy cases are underrated in rural and peri-urban settings and may limit the best practices for diagnosis and treatment. These challenges may influence the burden of acquired epilepsy in the study area and elsewhere in the country. These findings align with a study not specifically KAP-based, which reported that medical professionals in primary healthcare facilities had limited diagnostic capability on NCC [9].

We observed that 66.2% of HCWs were not aware of the recommended drugs for the treatment of NCC in PWE. This indicated a significant gaps in practices management regarding NCC-induced epilepsy and epileptic seizures in primary healthacare settings. Limited awareness and poor prescription of the recommended drugs among HCWs could negatively impact the diagnosis and treatment of NCC in PWE. With local health centers without neuroimaging facilities, they don’t get to diagnose NCC and therefore may not prescribe the appropriate treatment. Furthermore, in regions where NCC is endemic, HCWs may not have access to the latest treatment guidelines. This could be the reason that the recommended drugs (praziquantel, albendazole, or a combination of them), and supportive drugs such as anti-inflammatories, anti-edema, and analgesics to manage PWE due to NCC were not well known among primary HCWs. Management of complex conditions like NCC requires better diagnostic training and access of the latest treatment regimens for HCWs, especially in areas with limited access to specialized care.

Medical technologies have advanced day to day; therefore, continuing medical education programs in primary healthcare facilities should be encouraged to enhance optimistic practice patterns and enable the HCWs to acquire and master new practice skills [43] such as efficacious drugs or new drug combinations, new diagnostic skills (diagnostic techniques and criteria), and treatment guidelines. In addition, the present study observed that half of the HCWs were aware of the use of anti-seizures to manage epileptic patients. However, frequent prescription of anti-epileptic drugs (AEDs) to undiagnosed PWE or without knowing the cause of such epileptic seizures may predispose to antiepileptic drug resistance (AEDR).

In the case of PWE due to NCC, epileptic seizures arise after the *T. solium* cysticerci start to degenerate in the brain tissues, which takes a long time and depends on the number and evolutive stage of cysts, location, and level of patients’ immunity [42]. This may cause the patients to experience periodic epileptic seizures or asymptomatic NCC. Due to the existence of such mechanisms, there is a possibility that PWE due to NCC may develop AEDR once they are admitted for continuous anti-seizure medication without resolution of *T. solium* cysticerci. Considering that NCC is prevalent in Tanzania, PWE should be screened for NCC, which is a treatable disease with cheap drugs (praziquantel, albendazole, or a combination of the two) that are easily accessible in primary health facilities. Therefore, proper diagnosis of NCC in PWE is critical for bridging the treatment gaps for acquired epilepsy in the country.

Moreover, public health intervention programs aimed at preventing, controlling, and eradicating endemic poverty-related and/or foodborne-related zoonotic diseases in many endemic areas should target both communities and HCWs in place. The knowledge gap on these particular diseases, NCC and NCC-induced epilepsy included, is considerable. Negative beliefs, attitudes, or perceptions of the communities may have an indirect effect on the attitudes and practices of the HCWs in rural settings that could be influenced by the adoption of cultural norms and interactions with inhabitants with cultural beliefs.

Modifying the attitudes of both communities and primary HCWs is very critical. It would create a fundamental foundation for good practice management and enhance good linkage between the communities and primary health facilities. This may reduce the treatment gaps for PWE due to NCC in the country and elsewhere in endemic NCC.

Community-based, knowledge-attitude-practices studies conducted on *Taenia solium* Cysticercosis and Taeniasis (TSCT), NCC, and/or epilepsy indicated limited knowledge of *T. solium* NCC infection and the association between NCC and epilepsy, adverse practices, and negative attitudes in Tanzania [10] and in Uganda [32]. These findings are almost similar to our current healthcare professional-based KAP study. Therefore, our study results are useful and can be generalized for proper planning and developing of sustainable public health intervention strategies for the control and eradication of neglected or poverty-related zoonotic diseases (NCC included) in other parts of Tanzania, and elsewhere in endemic settings.

### Conclusion

Our study established knowledge gaps, negative attitudes, and poor practices about the prevention and control of NCC-induced epilepsy among HCWs in northern Tanzania. This could have led to a substantial burden of acquired epilepsy cases in the study area and possibly other parts of the country, creating barriers to the prevention and control or eradication of NCC and other endemic zoonotic diseases in Tanzania. Recognizing that NCC is a preventable cause of epilepsy, there is an urgent need to promote better knowledge of acquired epilepsy and epileptic seizures among primary HCWs who are confronted first with PWE. This would help to reduce epilepsy burdens and warrant health intervention programs regarding acquired epilepsy at the local context and national levels.

### Strengths and limitations of the study

On the strength of our study; we administered questions face-to-face to 250 HCWs in the selected primary health facilities to capture their knowledge, attitudes, and practices about NCC-related epilepsy.

On limitation; our study had limited time and resources, which makes it focused on only two districts, although there are other known districts to be endemic to *T. solium* NCC in the country. Also, we used a proportion-to-size sampling technique to obtain the number of HCWs by professional categories of which nurses were many compared to other categories in the study area.

## Supporting information

S1 Appendix Questionnaire Survey. (PDF)

S1 Dataset Numeric variables and String variables. (ZIP)

S1 Fig Geographical distribution of sampling sites in Manyara, Tanzania. The green colour indicates sampling points (Babati rural and Mbulu Districts). [Source: Generated from ArcGIS software, and the Basemap layer/shapefiles can be accessed at https://figshare.com/s/b7a45c4c7f85659da0bb.
(TIFF)

## References

[1] Stelzle D, Schmidt V, Ngowi BJ, Matuja W, Schmutzhard E, Winkler AS. Lifetime prevalence of epilepsy in urban Tanzania – A door-to-door random cluster survey. eNeurologicalSci. 2021;24. 10.1016/j.ensci.2021.100352 PMC8220170 34189286

[2] Owolabi LF, Adamu B, Jibo AM, Owolabi SD, Isa AI, Alhaji ID, et al. Prevalence of active epilepsy, lifetime epilepsy prevalence, and burden of epilepsy in Sub-Saharan Africa from meta-analysis of door-to-door population-based surveys. Epilepsy Behav. 2020;103. 10.1016/j.yebeh.2019.106846 31941583

[3] Makasi CE, Kilale AM, Ngowi BJ, Lema Y, Katiti V, Mahande MJ, et al. Knowledge and misconceptions about epilepsy among people with epilepsy and their caregivers attending mental health clinics: A qualitative study in Taenia solium endemic pig-keeping communities in Tanzania. Epilepsia Open. 2023;8(2):487–96. doi: 10.1002/epi4.12720 36896648 PMC10235557

[4] Millogo A, Kongnyu Njamnshi A, Kabwa-PierreLuabeya M. Neurocysticercosis and epilepsy in sub-Saharan Africa. Brain Res Bull. 2019;145:30–8. doi: 10.1016/j.brainresbull.2018.08.011 30170188

[5] Carod J-F, Dorny P. Cysticercosis in Madagascar. J Infect Dev Ctries. 2020;14(9):931–42. doi: 10.3855/jidc.13450 33031077

[6] Schmidt V, O’Hara M-C, Ngowi B, Herbinger K-H, Noh J, Wilkins PP, et al. Taenia solium cysticercosis and taeniasis in urban settings: Epidemiological evidence from a health-center based study among people with epilepsy in Dar es Salaam, Tanzania. PLoS Negl Trop Dis. 2019;13(12):e0007751. doi: 10.1371/journal.pntd.0007751 31809501 PMC6897529

[7] Herrador Z, Pérez-Molina JA, Henríquez Camacho CA, Rodriguez-Guardado A, Bosch-Nicolau P, Calabuig E, et al. Imported cysticercosis in Spain: A retrospective case series from the +REDIVI Collaborative Network. Travel Med Infect Dis. 2020;37. 10.1016/j.tmaid.2020.101683 32335208

[8] Bustos J, Gonzales I, Saavedra H, Handali S, Garcia HH. Neurocysticercosis. A frequent cause of seizures, epilepsy, and other neurological morbidity in most of the world. J Neurol Sci. 2021;427. 10.1016/j.jns.2021.117527 PMC8800347 34147957

[9] Mlowe F, Karimuribo E, Mkupasi E, Churi A, Nyerere AD, Schmidt V, et al. Challenges in the Diagnosis of Taenia solium Cysticercosis and Taeniosis in Medical and Veterinary Settings in Selected Regions of Tanzania: A Cross-Sectional Study. Mancianti F, editor. Vet Med Int. 2022;2022:1–15. https://www.hindawi.com/journals/vmi/2022/7472051//. 10.1155/2022/7472051 PMC9262556 35815231

[10] Nyangi C, Stelzle D, Mkupasi EM, Ngowi HA, Churi AJ, Schmidt V, et al. Knowledge, attitudes and practices related to Taenia solium cysticercosis and taeniasis in Tanzania. BMC Infect Dis. 2022;22(1):534. doi: 10.1186/s12879-022-07408-0 35692033 PMC9190087

[11] Gabriël S, Dorny P, Mwape KE, Trevisan C, Braae UC, Magnussen P. Control of Taenia solium taeniasis/cysticercosis: The best way forward for sub-Saharan Africa?. Acta Trop. 2017;165:252–60. 27140860 10.1016/j.actatropica.2016.04.010

[12] Maganira JD, Kidima W, Mwita CJ, Halvarsson P, Höglund J. Soil contamination by Taenia solium egg DNA in rural villages in Kongwa district, Tanzania. Infect Ecol Epidemiol. 2020;10(1). 10.1080/20008686.2020.1772668 PMC7448889 32922689

[13] Segala FV, De Vita E, Amone J, Ongaro D, Nassali R, Oceng B. Neurocysticercosis in low- and middle-income countries, a diagnostic challenge from Oyam district, Uganda. Infect Dis Rep. 2022;14(4):505–8. 35893473 10.3390/idr14040054 PMC9326654

[14] Kayuni EN. Socio-economic and health costs of porcine/human cysticercosis, neurocysticercosis and epilepsy to small-scale pig producers in Tanzania. Bull Natl Res Cent. 2021;45(1):217. doi: 10.1186/s42269-021-00676-x 34924747 PMC8669219

[15] WHO. Taeniasis/cysticercosis. 2022.

[16] Nau AL, Mwape KE, Wiefek J, Schmidt K, Abatih E, Dorny P. Cognitive impairment and quality of life of people with epilepsy and neurocysticercosis in Zambia. Epilepsy Behav. 2018;80:354–9. 29221763 10.1016/j.yebeh.2017.10.042

[17] Pesantes MA, Moyano LM, Sommerville C. Neurocysticercosis in Northern Peru: qualitative insights from men and women about living with seizures. PLoS Negl Trop Dis. 2020;14(10):1–16. 10.1371/journal.pntd.0008715 PMC7577431 33035212

[18] Devi KR, Borbora D, Upadhyay N, Goswami D, Rajguru SK, Narain K. High prevalence of neurocysticercosis among patients with epilepsy in a tertiary care hospital of Assam, India. Trop Parasitol. 2022;12(1):15–20. doi: 10.4103/tp.TP_72_20 35923269 PMC9341139

[19] Assane YA, Trevisan C, Schutte CM, Noormahomed EV, Johansen MV, Magnussen P. Neurocysticercosis in a rural population with extensive pig production in Angónia district, Tete Province, Mozambique. Acta Trop. 2017;165:155–60. 26519884 10.1016/j.actatropica.2015.10.018 PMC6333921

[20] Mwape KE, Blocher J, Wiefek J, Schmidt K, Dorny P, Praet N, et al. Prevalence of neurocysticercosis in people with epilepsy in the Eastern province of Zambia. PLoS Negl Trop Dis. 2015;9(8):e0003972. doi: 10.1371/journal.pntd.0003972 26285031 PMC4540454

[21] Ngowi HA, Winkler AS, Braae UC, Mdegela RH, Mkupasi EM, Kabululu ML, et al. Taenia solium taeniosis and cysticercosis literature in Tanzania provides research evidence justification for control: a systematic scoping review. PLoS One. 2019;14(6). 10.1371/journal.pone.0217420 PMC6550401 31166983

[22] Hunter E, Burton K, Iqbal A, Birchall D, Jackson M, Rogathe J. Cysticercosis and epilepsy in rural Tanzania: a community-based case-control and imaging study. Tropical Med Int Health. 2015;20(9):1171–9. 10.1111/tmi.12529 PMC5496663 25940786

[23] Mwang’Onde BJ, Chacha MJ, Nkwengulila G. The status and health burden of neurocysticercosis in Mbulu district, northern Tanzania. BMC Res Notes. 2018;11(1). 10.1186/s13104-018-3999-9 PMC6293552 30545404

[24] Stelzle D, Makasi C, Schmidt V, Trevisan C, van Damme I, Welte TM, et al. Epidemiological, clinical and radiological characteristics of people with neurocysticercosis in Tanzania–a cross-sectional study. PLoS Negl Trop Dis. 2022;16(11). 10.1371/journal.pntd.0010911 PMC9704569 36441777

[25] Mwang’onde BJ, Nkwengulila G, Chacha M. The risk factors for human cysticercosis in Mbulu District, Tanzania. Onderstepoort J Vet Res. 2014;81(2):E1-5. doi: 10.4102/ojvr.v81i2.719 25005750

[26] The United Republic of Tanzania Administrative Units Population Distribution Report. 2022.

[27] Ngowi HA, Kassuku AA, Maeda GEM, Boa ME, Carabin H, Willingham AL 3rd. Risk factors for the prevalence of porcine cysticercosis in Mbulu District, Tanzania. Vet Parasitol. 2004;120(4):275–83. doi: 10.1016/j.vetpar.2004.01.015 15063938

[28] Kavishe MDB, Mkupasi EM, Komba EVG, Ngowi HA. Prevalence and risk factors associated with porcine cysticercosis transmission in Babati district, Tanzania. Livestock Res Rural Dev. 2017;29(1):13.

[29] Israel GD. Determining Sample Size 1 The Level Of Precision. 1992.

[30] Monje F, Erume J, Mwiine FN, Kazoora H, Okech SG. Knowledge, attitude and practices about rabies management among human and animal health professionals in Mbale District, Uganda. One Health Outlook. 2020;2:24. doi: 10.1186/s42522-020-00031-6 33829139 PMC7993504

[31] Kainga H, Mponela J, Basikolo L, Phonera MC, Mpundu P, Munyeme M. Assessment of knowledge, attitudes, and practices towards Rift Valley fever among livestock farmers in selected Districts of Malawi. Trop Med Infect Dis. 2022;7(8). 10.3390/tropicalmed7080167 PMC9415226 36006259

[32] Alarakol SP, Bagaya BS, Yagos WO, Odongo Aginya EI. Knowledge, attitudes and practices of communities towards neurocysticercosis in the districts of Amuru and Gulu, Northern Uganda. J Parasitol Vector Biol. 2020;12(2):17–28.

[33] Habib MA, Dayyab FM, Iliyasu G, Habib AG. Knowledge, attitude and practice survey of COVID-19 pandemic in Northern Nigeria. PLoS One. 2021;16(1):e0245176. doi: 10.1371/journal.pone.0245176 PMC7808653 33444360

[34] Ertel RL, Braae UC, Ngowi HA, Johansen MV. Assessment of a computer-based Taenia solium health education tool ‘The Vicious Worm’ on knowledge uptake among professionals and their attitudes towards the program. Acta Trop. 2017;165:240–5. 26536396 10.1016/j.actatropica.2015.10.022

[35] Mambo R. Gaps in management and treatment of epilepsy in people with confirmed or probable neurocysticercosis. Lusaka: University of Zambia School of Veterinary Medicine Department of Clinical Studies; 2020.

[36] Alberts A, Jeanne Publisher A. Healthcare Providers’ and Community Leaders’ Knowledge and Perceptions of Neurocysticercosis in Rural Bolivia Item Type text; Electronic Dissertation [Internet]. 2019. Available from: http://hdl.handle.net/10150/634389

[37] Ngwili N, Thomas L, Githigia S, Johnson N, Wahome R, Roesel K. Stakeholders’ Knowledge, attitude, and perceptions on the control of Taenia solium in Kamuli and Hoima Districts, Uganda. Front Vet Sci. 2022;9. 10.3389/fvets.2022.833721 PMC9021822 35464359

[38] Chomba EN, Haworth A, Atadzhanov M, Mbewe E, Birbeck GL. Zambian health care workers’ knowledge, attitudes, beliefs, and practices regarding epilepsy. Epilepsy Behav. 2007;10(1):111–9. doi: 10.1016/j.yebeh.2006.08.012 17055341 PMC2938019

[39] Buddhiraja R, Sharma S, Sharma S, Rajnder, Bansal K, Setia RK. Epilepsy knowledge, attitudes and practices among primary health care providers in an Indian district. 2020. 10.1016/j.yebeh.2019.106899 32058300

[40] Muhammad S, Kabir S. Positive Attitude Can Change Life [Internet]. 2018. Available from: https://www.researchgate.net/publication/325712867

[41] Millogo A, Ngowi AH, Carabin H, Ganaba R, Da A, Preux PM. Knowledge, attitudes, and practices related to epilepsy in rural Burkina Faso. 2019. 10.1016/j.yebeh.2019.03.006 PMC6686174 31026786

[42] Guzman C, Garcia HH. Current diagnostic criteria for neurocysticercosis. Res Rep Tropical Med. 2021;12:197–203. 10.2147/RRTM.S285393 PMC8364393 34408532

[43] Samanta D, Hoyt L, Perry M. Healthcare professionals’ knowledge, attitude, and perception of epilepsy surgery: a systematic review. Epilepsy Behav. 2021;122. 10.1016/j.yebeh.2021.108199 PMC8429204 34273740

