# Supplementary material for: Knowledge, attitudes, and practices towards prevention and control of neurocysticercosis-related epilepsy in Northern Tanzania: A cross-sectional healthcare professional-based study
Source: PLoS Negl Trop Dis. 2025 Aug 1;19(8):e0013275. doi: 10.1371/journal.pntd.0013275 (PMC12316252; doi:10.1371/journal.pntd.0013275)
Supplement: S1 Appendix — (PDF) [file pntd.0013275.s001.pdf]

QUESTIONNAIRE SURVEY

Aim: To establish Knowledge, attitude and practices towards prevention and control of neurocysticercosis (NCC)-induced epilepsy among healthcare workers in Babati rural and Mbulu districts, in Manyara Tanzania.

Introduction and Consent

Greetings,

Participation in this study is willingly, and we would also like to inform you that there is no risk to participating in the study and that everything that you share with me will be kept confidential.

Do you agree to take part in this survey? YES ☐ NO ☐

Signature________________________

Date of interview _____________________

PART 1: Socio-demographic characteristics [Put a tick (✓) to the appropriate answer]

a) Respondent's gender; Male ☐ Female ☐
b) Your age _________ years
c) Education level: Standard seven ☐ Form four ☐ Certificate ☐ Diploma ☐ Advanced diploma ☐ Degree ☐ Master's degree ☐
d) Professional category; Nurse (Nurse and Midwifery, Assistant Nurse officer, Nurse officer) ☐ Clinical assistant (CA) ☐ Clinical officer (CO) ☐ Community health worker ☐ Health laboratory technologist ☐ Assistant medical officer (AMO) ☐ Medical doctor (MD) ☐
e) Working experience; _________ years

PART II: Knowledge [Put a tick (✓) in appropriate answer(s)]

1. Have you heard about Neurocysticercosis (NCC)? YES ☐ NO ☐
2. Is it a zoonotic disease? YES ☐ NO ☐
3. What is the cause of Neurocysticercosis? Put a tick (✓) in appropriate answer

	YES	NO
Taenia saginata /beef tapeworm (Mnyoo tegu wa ng'ombe)		
Taenia solium /pork tapeworm (Mnyoo tegu wa nguruwe)		
Dogs' tapeworms (Minyoo tegu ya Mbwa)		

4. What do you understand by the term neurocysticercosis?

	YES	NO
A neurological condition occurs when the larval (cysticerci) of beef tapeworm infects the brain of humans		
A neurological condition occurs when the larval(cysticerci) of pork tapeworm *Taenia solium* infect the brain of humans		
A neurological condition occurs when the adult tapeworms *Taenia solium* lodge into the brain of humans		

5. What is involved in the transmission cycle of neurocysticercosis?

	YES	NO
Pigs only		
Both pigs and humans		
Cattle, pigs and humans		
Humans only		

6. How do people acquire the infection of Neurocysticercosis? Please tick all answers that apply

	YES	NO
Through eating raw or undercooked beef		
Through eating raw or undercooked pork		
Inherit from parents		
Through ingestion of pig tapeworms' eggs from food, fruits or water contaminated with human faeces (faecal-oral transmission)		
Through autoinfection especially during active ant-peristalsis		

7. Is there any relationship between neurocysticercosis and epilepsy? YES ☐ NO ☐
8. If yes in question7 above, how do they relate? Please tick all answers that apply

	YES	NO
Neurocysticercosis induce epilepsy		
Epilepsy and/or epileptic seizures dominate in patients with Neurocysticercosis		
Neurocysticercosis (NCC) contributes to disabilities		

9. What are the common symptoms in Neurocysticercosis patients? Please tick all answers that apply

	YES	NO
Epilepsy or epileptic seizures		
Chronic headaches		
Focal neurological deficits, and cognitive alterations		
Blindness		
Dizziness and dementia		

10. The onset of neurocysticercosis symptoms follows the degeneration of *Taenia solium* larval (cysticerci) in the brain of the patient? YES ☐ NO ☐
11. Do you think that Neurocysticercosis (NCC) can cause Epilepsy? YES ☐ NO ☐
12. Can neurocysticercosis in patients with epilepsy (PWE) be treated? YES ☐ NO ☐
13. Can neurocysticercosis-induced epilepsy be prevented and/or controlled? YES ☐ NO ☐
14. If yes in Qn 13 above, what are measures that can be employed? [Please tick all answers that apply]

	YES	NO
Improvement of personal hygiene and sanitary conditions		
Early detection and treatment of human tapeworm carriers using effective anthelmintics such as (albendazole, niclosamides and praziquantel)		
Proper pork handling at household and community levels		
Improved standards of meat inspection		
Proper pig management practices		
To stop feeding pigs human faeces		

Vaccination of pigs		
Community health education		
Eating properly cooked pork		
Treatment of infected pigs using effective anthelmintics		
Proper diagnosis and treatments of neurocysticercosis-induced epilepsy cases		

15. Does the prevention and control of Neurocysticercosis-induced epilepsy require the involvement of veterinary practices, environmental health and medical practices?
    YES ☐ NO ☐

PART III: Practitioners' attitudes [Put a tick (✓) in appropriate answer(s)]

16. Have you heard a disease called epilepsy? YES ☐ NO ☐
17. Epilepsy is an inherited disease or disorder from parents or ancestors? YES ☐ NO ☐
18. What is the trend of epilepsy cases at your facility?

	YES	NO
Increasing		
Decreasing		
I don't know		

19. Do you believe that the tapeworm *Taenia solium* can cause epilepsy? YES ☐ NO ☐
20. Do you believe that people who eat raw or undercooked pork are likely to acquire epilepsy? YES ☐ NO ☐
21. Do you believe that unhygienic use of toilets is a risk factor for acquiring Neurocysticercosis-induced epilepsy? YES ☐ NO ☐
22. Patients with epilepsy can be diagnosed? YES ☐ NO ☐
23. Can people with epilepsy be treated? YES ☐ NO ☐
24. If yes in Qn 23 above, what is the mode of treatment? Tick all that apply

	YES	NO
Traditional treatments		

Modern treatments (Chemotherapy)		
Both traditional and Modern treatments		

25. Which of the following drugs do you believe to give your patients with epilepsy? Tick all that apply

Drug	Tick
Phenobarbitone	
Carbamazepine	
Phenytoine	

PART IV: Health professionals' practice [Put a tick (✓) in appropriate answer(s)]

26. Have you meet with patient with epilepsy? YES ☐ NO ☐
27. Do you manage patients with epilepsy at your facility? YES ☐ NO ☐
28. How many patients with epilepsy do attend per month? 1-5 ☐ 6-10 ☐ 11-20 ☐ above 20 ☐
29. Can epilepsy be diagnosed? YES ☐ NO ☐
30. If yes in Qn 29 above, where is the diagnosis done?

	YES	NO
At home		
At dispensaries		
In the community		
At the health centers		
At the hospital		

31. Do you consider Neurocysticercosis (NCC) as part of differential diagnosis for epilepsy cases? YES ☐ NO ☐
32. When you diagnose epilepsy at your facility, what do you do?

	YES	NO
Refer to traditional healer		
Treat		
Refer to higher hospital		
Do nothing		

33. Do you diagnose Neurocysticercosis-induced epilepsy? YES ☐ NO ☐
34. If yes in question 33 above, what are the diagnostic techniques do you use?

	YES	NO
Magnetic resonance imaging (MRI)		
Computed tomography (CT) scanning		
Immunodiagnostic techniques such as Enzyme-linked immunosorbent Assay (ELISA), Enzyme-linked immunoelectrotransfer blot (EITB)		
There are no specific diagnostic techniques		

35. Diagnosis of Neurocysticercosis in patient with epilepsy (PWE) is mainly based on neuroimaging techniques? YES ☐ NO ☐
36. Do you know a standard chart of diagnostic criteria for Neurocysticercosis (NCC) in patient with epilepsy (PWE) which is widely used worldwide? YES ☐ NO ☐
37. Do you provide a referral for patients with epilepsy for further diagnosis and treatments? YES ☐ NO ☐
38. Can neurocysticercosis-induced epilepsy be treated? YES ☐ NO ☐
39. Do you use the national guideline or the WHO guideline for management of people with epilepsy/epileptic seizures due to Neurocysticercosis? YES ☐ NO ☐
40. If yes to question 39 above, what are recommended drugs for treatment of Neurocysticercosis in people with epilepsy? Please tick all answers applies to you

	YES	NO
Praziquantel		
Albendazole		
Antibiotics		
Combination of Albendazole and Praziquantel		
Ant-seizures		
Ant-inflammatory such as dexamethasone		
Analgesics		
Ant-edema such as mannitol		

41. Do you titrate the medication for your epilepsy patients? YES ☐ NO ☐

42. Do you make active follow-ups of the patients placed on treatment with antiepileptic drugs?
YES ☐ NO ☐

43. Do you demand that epileptic patients give you feedback on the treatment you have given?
YES ☐ NO ☐

44. Do you refer epileptic patients to a higher-level hospital for further diagnosis?
YES ☐ NO ☐

45. For patients on anti-epileptic drugs (AEDs), do you make treatment follow-ups? YES ☐ NO ☐

46. Do patients who are on anti-epileptic drugs (AEDs) report back to your facility? YES ☐ NO ☐

47. Do you know of any epilepsy patients that have come off treatment? YES ☐ NO ☐

48. If yes, to question 47 above, what reasons do they give for stopping treatment?

	YES	NO
Side effects		
Treatment not working		
Run out of drugs.		

49. What do you do for patients that have stopped treatment?

	YES	NO
Change the drug		
Change the dosage of the drug they are on		
Refers to higher hospitals		

50. Do you report a suspect case of Neurocysticercosis-induced epilepsy? YES ☐ NO ☐

51. If yes in question 50 above, how many cases per month? 1-4 cases ☐ 5-10 cases ☐ 11-20 cases ☐ Above 20 cases ☐

ENDS
