# Supplementary figures and images for: Knowledge, attitudes, and practices towards prevention and control of neurocysticercosis-related epilepsy in Northern Tanzania: A cross-sectional healthcare professional-based study
Source: PLoS Negl Trop Dis. 2025 Aug 1;19(8):e0013275. doi: 10.1371/journal.pntd.0013275 (PMC12316252; doi:10.1371/journal.pntd.0013275)

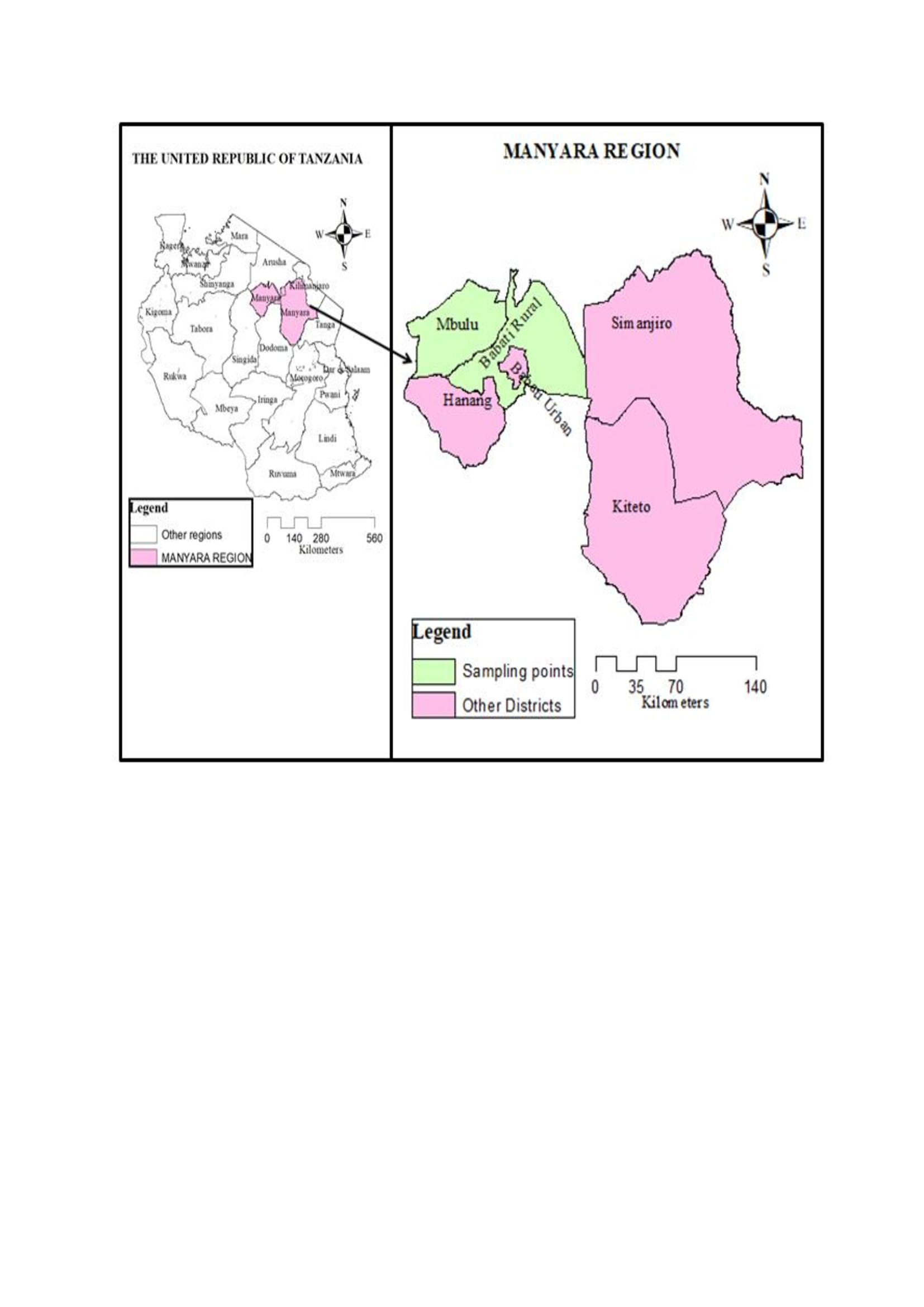

Supplement: S1 Fig — The green colour indicates sampling points (Babati rural and Mbulu Districts). [Source: Generated from ArcGIS software, and the Basemap layer/shapefiles can be accessed at https://figshare.com/s/b7a45c4c7f85659da0bb. (TIFF) [file pntd.0013275.s003.tiff]
